# Image integrity and standards

**DOI:** 10.1098/rsob.200165

**Published:** 2020-06-24

**Authors:** Jon Pines

**Affiliations:** *Editor-in-Chief*

**Keywords:** image integrity, screening, open biology

We have recently published Expressions of Concern for two papers in *Open Biology* that were identified as part of a group of eight papers with identical figures and tables. Should, as seems likely, these papers prove to be fraudulent then this strikes at the heart of our endeavour. Science depends absolutely on trust, and the vast majority of scientists honestly seek after the truth in their research; if errors are made these are honest mistakes in preparing figures or interpretation of data. But some errors can also be introduced by being too trusting, notably in the specificity of reagents, and, most regrettably, in the very small minority of studies where data are deliberately fabricated. Disturbingly, it appears that the number of fraudulent papers is on the rise, in part through authors purchasing papers through ‘paper mills' that generate papers by recycling text and figures. Thus, it is necessary for all publishers to increase their vigilance against deliberate fraud. The motto of the Royal Society is ‘Nullis in verba' (on the word of no one) and so it is appropriate that *Open Biology* should require of its authors that they provide rigorous data to support their conclusions. In this spirit, I would like to set out the standards required before publication, and the measures that we take with every paper to prevent fraud now and in the future.

— From now on, all gels and immunoblots will require molecular mass markers and for each figure, the whole gel or blot must be submitted as supporting data. Appropriate positive and or negative controls must be run on the same gel or blot, and the specificity of antibodies used for immunofluorescence should be validated by blotting or siRNA controls.
— For siRNA studies, at least three separate siRNAs must be used unless the phenotype can be restored by expressing an RNA resistant to the siRNA.
— All figure legends should state how many biological repeats were performed: a minimum of three is expected for most experiments.
— Images should not be subjected to any processing that could lead to misinterpretation of the original data, and the original, unprocessed data should be made available upon request.

To guard against deliberately fraudulent papers, all submitted papers will be screened using anti-plagiarism software and those provisionally accepted for publication will be examined by a third-party image integrity analyst. Any papers confirmed to contain plagiarized, manipulated or fraudulent data will be rejected and recorded in a database. Any published papers subsequently identified as potentially fraudulent will be treated according to the guidelines of the Committee on Publication Ethics.

More details can be found here: https://royalsocietypublishing.org/rsob/for-authors#question3.

I am sure that all our authors will understand that these measures are done to ensure that the community can trust in the validity of the papers published in *Open Biology*; it is only with this that we can all make progress.

## Supplementary Material

Reviewer comments

